# “I Want to See a Drowning-Free Philippines”: A Qualitative Study of the Current Situation, Key Challenges and Future Recommendations for Drowning Prevention in the Philippines

**DOI:** 10.3390/ijerph18020381

**Published:** 2021-01-06

**Authors:** Jonathan P. Guevarra, Richard C. Franklin, Amy E. Peden

**Affiliations:** 1 Department of Health Promotion and Education, College of Public Health, University of the Philippines Manila, 625 Pedro Gil St., Ermita, Manila 1000, Philippines; jpguevarra2@up.edu.ph; 2 College of Public Health, Medical and Veterinary Sciences, James Cook University, Townsville, Queensland 4811, Australia; a.peden@unsw.edu.au; 3 Royal Life Saving Society—Australia, Broadway, New South Wales 2007, Australia; 4 School of Population Health, University of New South Wales, Kensington, New South Wales 2052, Australia

**Keywords:** drowning, injury, epidemiology, policy, government, qualitative, interviews, stakeholder, safety, prevention

## Abstract

Drowning is described as a public health challenge by the World Health Organization. This challenge faces the Philippines where drowning claims an average of 3276 lives annually (3.50/100,000 population). However, no research comprehensively documents prevention strategies implemented by government and non-government agencies at a national or local level in the Philippines. This study aimed to qualitatively explore the current situation and key challenges of preventing drowning in the Philippines through key informant interviews and make recommendations to guide prevention efforts. Interviews were conducted among government and non-government agencies involved in drowning prevention using purposive sampling. Qualitative data collected were thematically analyzed. Findings indicate government and non-government agencies implement drowning prevention programs or strategies based on the mandate of their institutions. Most commonly cited were activities related to education or information awareness, emergency and disaster preparedness, and swimming skills. It was revealed that each agency was relatively unaware of the drowning prevention programs of other agencies. A multisector approach is needed to develop coordinated and comprehensive programs and activities aimed at preventing drowning. In this way, duplication will be avoided and the minimal resources available will be used effectively to reduce the burden of drowning in the Philippines.

## 1. Introduction

“*I want to see a drowning-free Philippines”—key informant*.

Drowning is the process of experiencing respiratory impairment from submersion or immersion in a liquid, with outcomes classified as mortality, morbidity or no morbidity [1]. It is estimated that 295,000 people died from unintentional drowning globally in 2017 [2], though this underreports the total burden due to exclusion of those drowning deaths due to transport-related incidents and disasters [3]. The World Health Organization (WHO) estimates that 45% of people who die globally from drowning are under 20 years of age [4]. Fatal drowning ranks as the 13th leading cause of death among children under 15 years of age, with the 1–4 years age group at greatest risk [2]. The overall global rate for drowning among children is 7.2 deaths per 100,000 people, although with significant regional variations [5]. The drowning rate in low- and middle-income countries (LMIC) is six times higher than in high-income countries (with rates of 7.8 per 100,000 and 1.2 per 100,000, respectively) [5].

**Figure 1:**
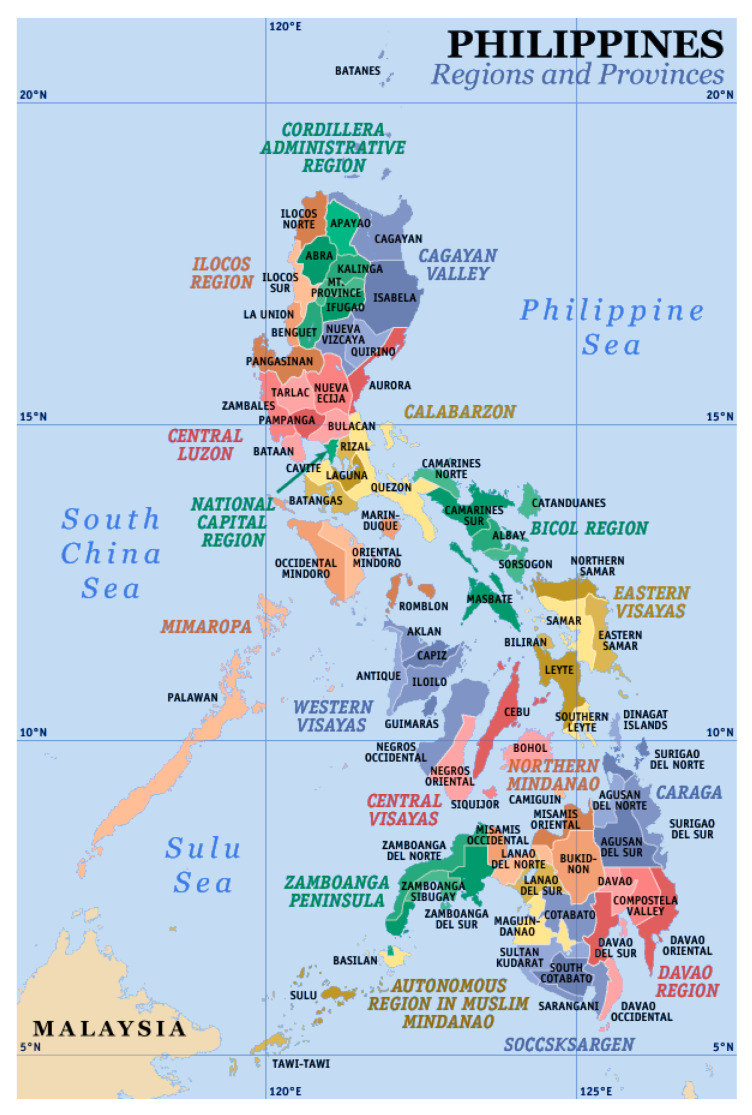
Map depicting the Philippines regions and provinces. © Eugene Alvin Villar, 2003 Reproduced under Creative Commons Attribution-Share Alike 3.0 Unported License [8].

The Philippines is a LMIC located in the Western Pacific region. It is a nation of 17 regions, 81 provinces, 144 cities, 1491 municipalities, and 42,028 barangays (villages), the smallest political unit in the Philippines. Covering an area of 343,448 square kilometers and with a population of 108,116,615 in 2019 [6], the Philippines is an archipelago comprised of 7107 islands. (Figure 1) Due to the nation’s island-based geography, interaction with water is commonplace with significant water-based transportation between islands and water recreation-based tourism [7]. 

On average, more than 3000 Filipinos of all ages died annually between 1963–2003 from drowning (0.43% of all deaths) [9]. Between 2006 and 2013, an average of 3276 deaths due to accidental drowning and submersion occurred in the Philippines per year, a rate of 3.5 per 100,000 population [10]. Among the fatalities, accidental drowning and submersion while in natural water was the main cause of recorded death [10]. Drowning data from the Global Burden of Disease study indicates 5245 people (95% Uncertainty Interval [UI]: 4635 to 5970) died from unintentional drowning in the Philippines in 2017, an increase of 5.4% (95%UI: −10.3% to 25.1%) on the 4977 drowning deaths recorded in 1990 [2]. Similarly, in 2017, the age-standardized cause-specific mortality rate for drowning was 5.3 per 100,000 population (95%UI: 4.7 to 6.0), a reduction of 28.6% (95%UI: −38.0% to −17.0%) from the rate of 7.4 (95%UI: 6.8 to 8.0) recorded in 1990 [2]. 

A third (35.6%) of all drowning deaths in the Philippines occur among children under 14 years of age [9]. Mortality is highest among children ages 1–4 years accounting for 5% of all deaths in this age group. Drowning mortality rates have remained largely unchanged from 1963–2003, belying its under recognition as a public health priority. From this epidemiological work, there was a recommendation for better surveillance of child drowning deaths to guide policy formulation for its prevention and treatment nationally [9].

In 2006, the Administrative Order No. 2006–0016 [11], also known as the “National Policy and Strategic Framework on Child Injury Prevention”, was enacted. Following its enactment, the Department of Health (DOH) of the Philippines began to build a national program on violence and injury prevention. This policy centered on planning interventions and strategies on violence and injury prevention for children. Priority areas included road traffic injuries, drowning, falls, burns and scalds, and poisoning [11].

From 2009–2010, the Philippines′ Department of Health (DOH), with the support of the WHO, selected pilot sites in Northern Philippines to deliver prevention programs targeting drowning. In 2011, an expansion site was selected in Northern Philippines, for another drowning prevention project. As part of the projects in these pilot sites, a community assessment determining the risk factors related to drowning was undertaken. Results from the assessment were presented to community stakeholders to obtain feedback. Planning and implementation of the community interventions then followed such as: the development of barriers (for porch and house); posting of drowning prevention messages; information dissemination; CPR training; basic swimming; and skills, were implemented at the village level and aimed to reduce drowning incidents in these areas [12]. These interventions for preventing drowning were based around the four stratagems of (1) legislation or the setting of rules (including enforcement); (2) public education and skill development, including improved ergonomic design, engineering and environmental measures; (3) combined approaches where people are saved subsequent to drowning such as the use of rescue and cardio pulmonary resuscitation [13]; and (4) provision of psychosocial support, all of which have been identified to reduce drowning [14].

Data from these interventions indicate that community pilot sites were receptive to the drowning prevention projects. Partner agencies such as Department of Health (DOH), the Local Government Unit (LGU), the City Health Office (CHO), the village leadership and WHO were instrumental in helping communities understand the need for such a project and to implement the proposed interventions. The drowning prevention program included the construction of porch barriers and backyard fences in homes built above or near water and the provision of playpens to physically prevent children falling into water. It also focused on rebuilding and covering wells. First aid training was provided to local officials, health workers and community volunteers so they could react better when children did fall into water. In one site, a Community Drowning Prevention Committee was created, composed of subcommittees on health; infrastructure; and monitoring and evaluation. The pilot project does not yet cover enough households to provide a measurable decline in drowning, however an awareness of drowning risks has increased. Families are willing to have barriers constructed; and the barriers themselves appear to be holding up well. Equally important, people looking after children recognize that the barriers will not prevent all accidents: constant supervision remains necessary [15].

To date, there is limited research which documents interventions for preventing drowning which are being implemented by government and non-government agencies at the national level in the Philippines. As such, this research aimed to investigate the current situation and identify future challenges for drowning prevention and water safety (henceforth refereed to collectively as drowning prevention) in the Philippines. Specifically, this study sought to (1) explore what information is currently available in the Philippines in relation to drowning prevention policy, programs, and research; (2) determine drowning prevention strategies currently being implemented in the Philippines; and (3) identify the challenges and opportunities in the implementation of drowning prevention efforts in the Philippines.

## 2. Materials and Methods

This study employed a qualitative approach using key informant interviews to collect information on drowning prevention activities and challenges. Interviews were conducted with key informants from government and non-government agencies involved in drowning prevention programs at the national level. This included organization whose primary focus was child injury prevention program (drowning prevention included) and other agencies with a broader remit which intersected with drowning prevention (i.e., maritime groups and organizations working in the field of emergency and disasters and local government).

### 2.1. Sampling Design and Sample Size

Purposive sampling was undertaken to ensure all key informants were interviewed. Key informants were identified from the Philippines Drowning Prevention Plan 2010-15 [16] (developed and spearheaded by a non-government organization) and a snowballing technique was then used to find other participants. Only informants from agencies at the national level were included. Key informants were selected and recruited based on the following criteria: (1) employee of agencies with the potential to undertake water safety activities (2) involved in the implementation of a drowning prevention program in the Philippines, and (3) Consented to the interview. Key informants were recruited via a letter. If no response was received, respondents were contacted a further two times via letter seeking their participation. 

### 2.2. Data Gathering Procedure

Permission to conduct the study was obtained from the relevant government and non-government agencies. Upon obtaining permission, the researcher explained the purpose of the study, risks and benefits to the informants. An informed consent form was signed prior to the interview. Permission to record the interview was also obtained. After turning over the signed informed consent form the interview commenced and conducted by the Filipino author (JPG). Interviews were semi-structured in format and used a key informant interview guide (Table 1). While data saturation was not reached within the interviews conducted, by the last interview there was only one new issue identified.

### 2.3. Data Analysis

Interviews were tape-recorded, transcribed and where required translated to English. Thematic analysis was conducted for the qualitative data collected from the interviews using an inductive approach as outlined by Braun and Clarke [17]. This five-phase methodology was undertaken as follows: Authors JG and RF familiarized themselves with the data by rereading the data and noting down initial themes (Phase 1). Authors JG and RF then generated initial themes by systematically coding interesting features of the entire dataset (Phase 2). Both authors then came together to cross-check and confirm individual thematic coding of themes (Phase 3). Separately, both authors sorted qualitative data into themes (Phase 4). Both authors then came back together to compare the contents of each theme and refine any outstanding issues (Phase 5) [17].

To ensure of trustworthiness of this qualitative research, that is, credibility, dependability, conformability, transferability and authenticity, the following steps were undertaken (1) the primary author (Filipino) conducted the interviews as well as the transcription of audio recorded data, and translation of the answers in the native language to English language. (2) Another co-author (RCF) reviewed the transcribed data. Analysis of the themes were reviewed by two authors independently (JPG and RCF) for accuracy (based on what was said during the interviews) and then compared. (3) To ensure that we captured what the interviewees have communicated to the interviewer, we returned again and again to the data, to check whether the interpretation is true to the data. 

### 2.4. Technical and Ethical Approval

This study obtained technical approval the College of Public Health, University of the Philippines Manila (CPH Technical Approval No. 2013–008) and ethical approval from the University of the Philippines Manila-Research Ethics Board (UPMREB 2013-130-01).

## 3. Results

Originally, eight key informants were purposively selected as respondents for the study. However, only four informants participated in the study. These informants (three males, one female) were from national government agencies (3) and non-government organization (1).

### 3.1. Thoughts on Drowning Prevention

All participants recognized the need to employ drowning prevention programs to ensure people’s safety from drowning. They highlighted the fact that people in the community are continually exposed to bodies of water due to the geographical features of the country and the Philippines being an archipelago. They identified that the risk of drowning increased as people crossed these bodies of water, or when there were emergencies like flooding, and that while swimming skills were useful, people were still at risk. They acknowledged that death due to drowning is highly preventable. As such, drowning prevention is vital to put an end to the unnecessary deaths due to drowning. This research was unable to find any country-wide drowning specific program. Most programs included drowning as part of a more general prevention program for other issues such as injuries. Nevertheless, there remains hope to prevent drowning and its societal consequences. As one informant said, “*…I want to see a drowning-free Philippines…*”.

### 3.2. Main Drowning Prevention Activities

The participating organizations’ major activities in preventing drowning in the community, included capacity building, education, skills training (for both their personnel and the broader community), community assessment of risks, information dissemination, injury prevention, use of materials to build structures for the physical prevention (i.e., barriers) of unintentional drowning, rescue operations especially in cases of disasters, as well as impact assessment of their drowning prevention activities and programs. The most commonly described interventions were at the community level and focused on knowledge improvement, skills training (basic swimming skills, CPR), and prevention of drowning through safety measures such as building barriers to prevent children accessing water.

#### 3.2.1. Physical Prevention of Drowning

**Figure 2:**
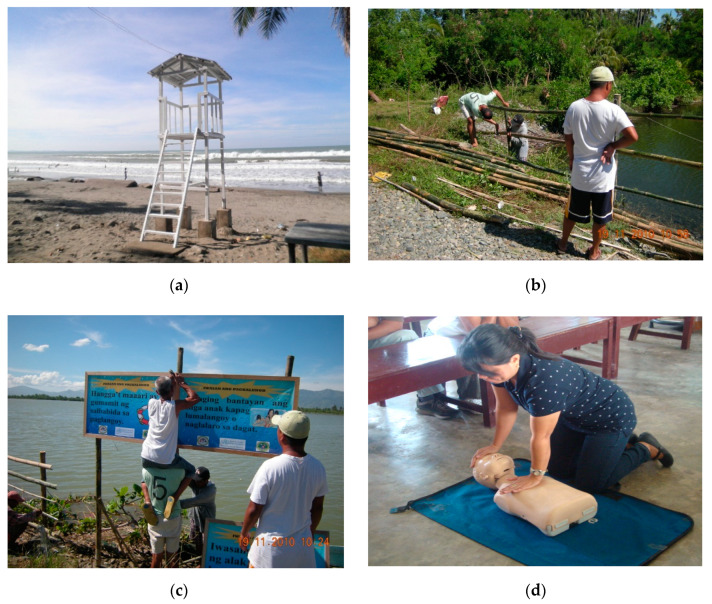
Community drowning prevention initiatives mentioned by key informants in the Philippines: (**a**) Guardhouses; (**b**) Barriers; (**c**) Signs; (**d**) Cardiopulmonary resuscitation (CPR) training.

A major activity undertaken by one organization through its pilot project was the installation of barriers in homes where the highest risk of drowning was identified. For high-risk coastal areas guardhouses were installed where a lifesaver manned the guardhouse to perform rescues or prevent people from entering the water (Figure 2). This informant mentioned “*…We also mobilized the village (barangay) to monitor the shore every night. We provided them megaphones and flashlights. They would also take turns in guarding the shore…*”. Barriers were installed where there had been incidents of drowning observed. Signs to warn people of drowning especially at high drowning locations (i.e., areas of previous drowning deaths) were also installed. For another organization, the key informant mentioned installing safety lines in coastal areas to ensure that people in the community are kept from unsafe areas.

#### 3.2.2. Capacity Building, Education and Skills Training

Skills training was another key activity undertaken, for both the community and their organization’s personnel. These activities focused on the capability to identify drowning individuals, swimming to and returning drowning individuals to shore, utilization of equipment in rescue operations, as well as basic life support for drowning individuals (cardiopulmonary resuscitation (CPR)). Skilled personnel from the organizations and their partner civilian organizations provided the training across the country.

One organization provided an extensive and sequential approach in its training program starting from swimming courses, to life saving courses, and its highest level, lifeguarding courses. These courses are available according to age and are further subdivided into basic swimming, advanced beginner swimming, intermediate swimming, and advanced swimming, and levels 1, 2, and 3 for its lifeguarding courses. Instructor courses were also being offered by the organization for more advanced participants.

Another informant shared that they assess the swimming capability of their trainees before they can graduate. Specific terms used by this informant were: “*…We conduct schooling… you cannot graduate unless you know how to swim. It’s already part of our preventive measures from drowning…*”

#### 3.2.3. Situational Assessment

Situational assessment was undertaken by two organizations before starting any intervention. An informant shared that their organization spearheaded the development of paper-based assessment tools (checklists) for school, home and the community. These assessment tools were used to assess the risk factors for injury in these settings. The key informant identified that one aspect of the tool assesses vulnerability of children to drowning. An informant mentioned that “*…In the past few years, we had our initiative of creating assessment tools which we used to assess drowning-prone in the different settings namely at school, in the community and at home. Drowning is included in that tool given that there are a number of infants and toddlers who were reported to have experienced drowning in the community, in the nearby lakes and rivers…*”

#### 3.2.4. Information Dissemination

Increasing the level of awareness of the public was one of the major activities covered by all organizations of the key informants interviewed. These activities were about the dissemination of the program, using information, education and communication (IEC) materials like signage, flyers, and flipcharts on child safety tips. Examples of messages that focused on drowning prevention were, “*…Never leave the baby alone while giving a bath…*”; “*…Empty water containers when not in use…*”; “*…Cover wells, manholes and drainages…*”; and “*…Wear personal flotation devices while swimming…*”

#### 3.2.5. Rescue Operation

The organizations of two key informants interviewed were involved in rescue operations. Whenever there is a national emergency and when a civilian agency or sector requests their assistance, they send their equipment and personnel to help. The informant from a government organization said “*…It is not just the national government but also the local government. We are qualified to conduct search and rescue…*” In many instances, these two organizations were being called to respond whenever there is flooding in the National Capital Region. They also mentioned that their efforts were being coordinated with the National Disaster Risk Reduction Management Council (NDRRMC), Department of Health (DOH) and the Philippine Coast Guard (PCG).

### 3.3. Challenges and Measures to Address the Challenges

Key informants mentioned a number of challenges they have experienced in the performance of their drowning prevention activities. These included issues with coordination and communication among organizations (lack of unity, and duplication of responsibilities), inadequate resources (fast turnover of personnel, inadequate skills, insufficient funds, lack of manpower, inadequacy of resources and materials), geographical difficulties, lack of information and data, and a lack of monitoring measures.

#### 3.3.1. Coordination and Communication among Organizations

All key informants shared the activities that their organizations implement. However, it was noted that there was no synergy in the implementation of these activities between organizations. Each organization implements its own programs and activities and in some instances key informants noted some duplication of efforts with other organizations. One informant lamented this, stating that, “*Sometimes when we meet, everyone even tries to boast about their own programs which I think is not good. In my opinion, we all have the same goal and that is to save life. We should not use our programs to make our organization or company look superior over another…*” When it comes to statistics, the informant added “*…You can only find answers in the World Health Organization. We should also have these statistics in our own country. That’s a clear indication that we are not united. We lack synergy in our work...*”

The informants mentioned that issues of fragmentation and non-unity can be addressed by harmonizing the policies and by having a clear set of responsibilities per agency. Having a fixed and permanent organizational structure would also help in addressing the problems of coordination and communication. One key informant specifically mentioned that “*…Through the united effort of different individuals and organizations, we can speed up the development that we want to happen. So, eventually, we will achieve a drowning-free Philippines if only we will help one another in implementing this program…*”

#### 3.3.2. Inadequate Resources

Resources mentioned by the key informants covered human, material and financial resources. For human resources, they mentioned either lack of or inadequate personnel, inadequate skills and fast turnover of personnel (“*…change of personnel handling the program…*” and “*…change of administration due to rationalization…*”). Key informants were also concerned about the inadequacy of resources in their organization and the insufficient funds they had compared to the funds needed to maintain the operational efficiency of their organization. One informant mentioned that “*…the national headquarters personnel cannot do the job by themselves going to every chapter from province to province…*” Another informant lamented that “*…Budget is very small. It is not even solely allotted for drowning but for child injury prevention programs…*” Informants recommended that government agencies must allocate a specific amount of budget for injury prevention in general and drowning prevention in particular. Another informant even suggested that funds be allocated for the conduct of research. The need to inculcate a culture of preparedness among the citizens of the Philippines was also identified. This may include preparedness in terms of materials and equipment which can be used in times of emergency.

#### 3.3.3. Geographical Difficulties

The Philippines is composed of many islands and different bodies of water can be found throughout the country. Implementing a comprehensive program covering the whole country was thought to be difficult by some key informants. One key informant specifically said that “*…There are LGUs located in the mountainous areas while others are almost surrounded by bodies of water. Because of this, implementing a program through a policy on the national setting could be difficult…*” Given this observation, informants stated it would still be helpful if there was a national policy on drowning prevention, however, prevention strategies should consider the diverse landscape of the Philippines and give consideration to the many bodies of water in both the inland and coastal areas (including man-made bodies of water).

#### 3.3.4. Lack of Information and Data

Just like in other programs, information and data about drowning are scarce, which also affects the development and implementation of a comprehensive program on drowning prevention. An informant said “*…I don’t know if there are agencies which can answer the statistics as to how many drowning incidents happen in year or a month…*”

#### 3.3.5. Lack of Monitoring Measures

One informant mentioned about monitoring of drowning activities and said “*…We were not able to monitor it {the project]…*” The issue about monitoring was apparent when another informant commented “*…Actually, I thank you that you have this [study] which monitors this area {drowning] because this has been neglected and no one takes action…*”

## 4. Discussion

This research provides a point-in-time exploration of responses to drowning in the Philippines and provides a baseline examination prior to the implementation of more substantial drowning prevention activities and the development of the multisector action plan (MSAP) [10]. While the response rate was low, the rich information discovered informed the development of the MSAP and the participating agencies of this study were also were involved in the development of the MSAP. The responses of the key informants interviewed identified activities on drowning prevention currently being implemented by each agency. These activities included situational assessment, capacity building, information dissemination, implementation of physical structures to prevent drowning, and rescue operation. It is likely that these activities do not represent a complete picture of all drowning prevention activities since only a handful of key informants and organizations participated in this study. Nevertheless, this study provides important points for future drowning prevention efforts in the Philippines.

Challenges surrounding the implementation of drowning prevention activities were identified which need to be addressed in order for drowning prevention programs in the Philippines to be successful. One of these challenges included coordination and communication with agencies involved in the implementation of injury prevention. There is a need for government, non-government and civil society groups to sit at one table and discuss how they can have a concerted effort in addressing the neglected public health issue of drowning in the Philippines. There is also a need to craft a national agenda that will harmonize the efforts of key players in the field of drowning prevention.

Different organizations need to allocate resources in order for drowning prevention efforts in the Philippines to be successful. In the event that a multisector group is formed, key players in this group can share their expertise and resources in order to have a unified method of collecting information/data on drowning in the country. This group can also develop strategies to effectively monitor efforts aimed at reducing the burden of drowning in the Philippines. Drowning is a not a problem of the health sector alone [18]. Different agencies have a stake in solving this issue. Transfer of information and skills are always a problem for country level programs and represent an area where future research would benefit not only drowning prevention but all programs being implemented at a national level across the Philippines.

The World Health Organization in its Global Report on Drowning (2014) recommended that all governments develop their own Drowning Prevention Plan through a multisector group [19]. A national drowning prevention plan has multiple impacts, however in the first instance the two most important would be: first, to bring all organizations involved in drowning prevention together to discuss what is needed to be done and secondly, to outline what this group feel are the priorities for drowning prevention in the Philippines. The next challenge once this had been achieved would be to resource the plan and continue the conversations started in the development of the plan.

In a document from the World Health Organization in the Western Pacific Region entitled “Regional Action Plan for Violence and Injury Prevention in the Western Pacific (2016–2020)”, it was recommended that the multi sectoral group must be composed agencies such as the children’s affairs, coast guard, disaster response, education, finance, health, planning and development, police and transport [20]. Such a group is needed both as drowning (and its prevention) is diverse and cross-sectoral [17], but also to address the lack of cohesion, poor communication and duplication of efforts and energy across those working in drowning prevention in the Philippines who were interviewed for this study.

More broadly, there were a range of other challenges identified by key informants in reducing drowning in the Philippines. These included inadequate resources, a lack of monitoring measures, and a lack of data and information on drowning and geographical difficulties. With respect to a lack of data and information, quality drowning data underpins evidence-informed prevention efforts [21] and has been identified by the WHO as a key element of a best-practice approach for preventing drowning [22]. There is an urgent need to establish a national drowning surveillance system that captures demographic and causal data on fatal and non-fatal drowning in the Philippines and a process to make de-identified data available to researchers. Investment in similar contexts, such as Bangladesh [23] and Thailand [24], has resulted in strengthened research and prevention efforts. Such a system will allow for the evaluation of interventions implemented, as well as the identification of emerging threats.

The geographical difficulties, and thus the diversity of water bodies posing a risk of drowning, was also identified by key informants as a challenge facing drowning prevention efforts in the Philippines. In particular, key informants identified that such difficulties may make the development of a national-level strategy to address drowning difficult. This underscores the need for local identification, ownership and implementation of community-support strategies to reduce drowning. This may include a situational risk assessment with community and co-design of a program, as has been done to great success in the provinces of Thailand through the community drowning surveillance work [24] and the MERITMAKER child drowning prevention intervention [25]. Similarly, low-cost interventions which utilize the community’s natural bodies of water for teaching children to swim, while also providing employment for adults, has been found to be an effective and community accepted approach to reducing child drowning in Bangladesh [23]. While an overarching national multisector action plan on drowning prevention is recommended to generate awareness on the issue with national-level stakeholders, a subsequent phase may be the development of localized action plans. The experiences of countries such as Thailand and Bangladesh may provide guidance to the Philippines as it aims to address similar challenges in reducing drowning.

### 4.1. Recommendations

In light of the research findings, and in accordance with recommendations made by the WHO to guide country-level drowning prevention efforts [19,20], the following recommendations are made to help prevent drowning in the Philippines: (1) Develop a national multisector plan on drowning prevention. This will address the fragmented approach to program prevention in the country. The individual effort of each organization involved in drowning prevention can be placed in one document that will be followed by all drowning prevention key players. (2) Form a steering committee. There are a number of positive points if a multisector group is formed. This group can be the steering committee that will be responsible in creating a comprehensive plan on drowning prevention. This group can also establish guidelines and standards on how to appropriately address drowning in the country. This group can also serve as the monitor and evaluator (with the help of experts) of drowning prevention initiatives in the country. The group can also document the best practices on drowning prevention which can be shared to different parts of the country and the rest of the world. (3) Conduct research on drowning and its prevention. For future studies, using a mixed methods approach will assist in gaining a more comprehensive picture of drowning and drowning prevention activities in the Philippines. Although a qualitative method is, in itself, a rich source of data, utilizing both quantitative and qualitative approaches can further enhance understanding of drowning burden, risk factors, behaviors and effective drowning prevention interventions. Similarly, there is the need for a comprehensive data collection system on the incidence and risk factors contributing to fatal and non-fatal drowning cases in the Philippines to underpin this research to guide prevention efforts.

### 4.2. Limitations

This is the first study to explore drowning prevention activities in the Philippines and one of very few to explore organizational and policy responses to drowning [26,27]. However, one of the limitations of this study was the small sample size which limits the generalizability of results. All effort was made to ensure that the key informants interviewed were the most appropriate person at the organization with respect to knowledge about the drowning prevention program being implemented. While the researchers believed that the participants are the ones with knowledge and thus representative, it was possible but unlikely that the researchers may have missed key respondents. Several attempts were made in order to cover all identified key informants; however, these efforts did not contribute to the success of engaging these identified informants. Nevertheless, the answers by the included key informants provided valuable information on drowning prevention initiatives in the Philippines not previously uncovered. It is also likely that at the time drowning prevention was not seen as a priority for some of the key organizations and as such they did not see value in participating.

## 5. Conclusions

A multisector approach is needed in order to develop coordinated and comprehensive programs and activities aimed at preventing drowning in the Philippines. In this way, the program or activities of one agency will complement the efforts of other similar-minded institutions and hopefully reduce the burden of drowning.

## Figures and Tables

**Table 1:** Key Informant Interview Guide.

Section	Content
Demographics	Position Title
	Age
	Sex
	Interviewer
	Date of Interview
	Time Started
	Time Ended
	Location (Venue)
Warm Up and Explanation	Introduction
	Explain purpose of interview
	Explain procedure, ask permission for use of tape recording
	Confidentiality statement
	Informed consent
Top of Mind Association	What comes to your mind when the word Drowning Prevention is mentioned? *Probe: When only one term is given ask for clarification/elaboration.*
In-Depth Interview	What is the role of your agency in drowning prevention and water safety?
	What are your programs for drowning prevention and water safety? *Probe for the following:*
	*Who (agencies/sectors) is delivering them;* *Types of programs being delivered;* *Who is the target of the programs;* *Geographical coverage;* *Where future activities need to be delivered.*
	Who are your partners in the implementation of drowning prevention and water safety? *Probe: for the roles/tasks and responsibilities of these partner agencies.*
	How do you sustain the efforts that you have started? *Probe: for resources, support of Local Government Unit and other stakeholders.*
	What challenges did you encounter in the implementation of your drowning prevention program?
	How did you address these challenges?
Closing	Check field notes for any clarification needed
	Check topic guide for any question missed
	Thank informant for time spent and for participation.

## Data Availability

Qualitative data collected for this study can be provided to approved users by contacting jpguevarra2@up.edu.ph.
